# Pay-for-performance in disease management: a systematic review of the literature

**DOI:** 10.1186/1472-6963-11-272

**Published:** 2011-10-14

**Authors:** Simone R de Bruin, Caroline A Baan, Jeroen N Struijs

**Affiliations:** 1 National Institute for Public Health and the Environment, Centre for Prevention and Health Services Research, P.O. Box 1, 3720 BA Bilthoven, The Netherlands

## Abstract

**Background:**

Pay-for-performance (P4P) is increasingly implemented in the healthcare system to encourage improvements in healthcare quality. P4P is a payment model that rewards healthcare providers for meeting pre-established targets for delivery of healthcare services by financial incentives. Based on their performance, healthcare providers receive either additional or reduced payment. Currently, little is known about P4P schemes intending to improve delivery of chronic care through disease management. The objectives of this paper are therefore to provide an overview of P4P schemes used to stimulate delivery of chronic care through disease management and to provide insight into their effects on healthcare quality and costs.

**Methods:**

A systematic PubMed search was performed for English language papers published between 2000 and 2010 describing P4P schemes related to the implementation of disease management. Wagner's chronic care model was used to make disease management operational.

**Results:**

Eight P4P schemes were identified, introduced in the USA (n = 6), Germany (n = 1), and Australia (n = 1). Five P4P schemes were part of a larger scheme of interventions to improve quality of care, whereas three P4P schemes were solely implemented. Most financial incentives were rewards, selective, and granted on the basis of absolute performance. More variation was found in incented entities and the basis for providing incentives. Information about motivation, certainty, size, frequency, and duration of the financial incentives was generally limited. Five studies were identified that evaluated the effects of P4P on healthcare quality. Most studies showed positive effects of P4P on healthcare quality. No studies were found that evaluated the effects of P4P on healthcare costs.

**Conclusion:**

The number of P4P schemes to encourage disease management is limited. Hardly any information is available about the effects of such schemes on healthcare quality and costs.

## Background

Chronic diseases are the leading cause of disability and death in the Western part of the world [1]. Over the coming years, the prevalence of chronic diseases is predicted to increase as a result of the rapid aging of the world population and the greater longevity of people with chronic conditions [2,3]. Healthcare systems struggle with coordinating care to people with chronic conditions. The healthcare system, traditionally predominated by a re-active approach and consisting of highly specialized echelons, needs to be transformed to realize patient-centered chronic care in which problems like fragmentation, restricted self-management, and guideline non-adherence are limited [4,5].

To deal with this challenge, policymakers and professionals introduced disease management programs (also referred to as e.g. case management, integrated care, managed care, and shared care) to enhance quality and continuity of care for the chronically ill. In broad terms disease management refers to a patient-centered approach of coordinated multiple healthcare interventions that aim to structure chronic care to a specific patient group [4,6]. A model that is perhaps best known from an international perspective, and that was used for this study to make disease management operational, is the Chronic Care Model (CCM) of Wagner et al. [5,7]. This model can be considered as a guide towards improving management and co-ordination of chronic conditions. The model suggests that disease management ideally comprises six interrelated components (i.e. health care organization, community resources, self-management support, decision support, delivery system design, and clinical information system) and that integration of these components is the key towards improving chronic care management [4].

It is generally believed that disease management programs result into improved patient health outcomes and into healthcare cost savings. There is, however, a lack of conclusive scientific evidence supporting these suggestions [4,6,8,9]. Nevertheless, interest in pay-for-performance (P4P) as a tool to stimulate delivery of chronic care through disease management is growing. P4P is a payment model that rewards healthcare providers for meeting pre-established targets for delivery of healthcare services by financial incentives [10]. Based on their performance, healthcare providers receive either additional or reduced payment. The reasoning behind P4P schemes is, by either rewarding or punishing healthcare providers for their performance, to improve quality of care [11,12]. P4P schemes, also known as e.g. performance based incentive programs and quality incentive payment systems, should not be confused with funding of disease management. Funding refers to recompensing delivery of healthcare services by healthcare providers via retrospective (e.g. fee-for-service or bundled payment) or prospective (e.g. capitation) payment contracts.

To date, little is known about the use of P4P schemes to stimulate delivery of chronic care through disease management and whether such schemes actually affect healthcare quality and healthcare costs. The objectives of the present paper are therefore (i) to provide an overview of P4P schemes that are currently used to stimulate delivery of chronic care through disease management and (ii) to gain insight into the effects of P4P on healthcare quality and healthcare costs.

## Methods

### Study design and search strategy

A systematic review of the literature was performed for insight into P4P schemes aiming at stimulation of delivery of chronic care through disease management. We conducted our search in PubMed focusing on English language papers published between January 2000 and January 2010. A comprehensive search strategy was developed by a librarian of our institute to identify studies matching the following search terms (Medical Subject Headings): *case management, comprehensive health care, delivery of integrated health care, disease management, managed care programs, patient care management, patient-centered care, shared care, transmural care *and variations of the keywords *chronic disease, chronically ill, chronic illness, long term care *and specified chronic conditions. These search terms were combined with variations of the following search terms: *bundled payment, fee for services, health care reform, incentives, local partnerships, pay for performance, payment methods, practice based commissioning, physician incentive plans, prospective payment system, quality assurance*, and *reimbursement (mechanisms)*.

In addition to the electronic database search, relevant papers were identified through reference tracking and through a manual literature search on the internet. To obtain up-to-date information about the included P4P schemes, also websites of the P4P schemes and other relevant websites such as those of health insurers and Ministries of Health were consulted.

### Study selection

Two reviewers (JS and SdB) independently reviewed the papers extracted by the search for their relevance by screening their title and abstract. If found relevant by both reviewers, the full-text paper was retrieved. Any disagreement between the reviewers was resolved by consensus. Papers describing P4P schemes focusing on the implementation of disease management programs were included.

In line with earlier studies [9,13,14] also in this study the chronic care model (CCM) of Wagner et al. [5,7] was used to make disease management operational. The model suggests that disease management ideally comprises six interrelated components. Four components refer to the actual delivery of care by healthcare providers of a healthcare organization; 1. *self-management support *that helps patients and their families to obtain skills and confidence to manage their chronic condition (e.g. blood glucose monitoring) and assessment of problems and achievements on a regular basis; 2. *delivery system design*; focus on coordinated multidisciplinary collaboration between caregivers (i.e. multidisciplinary team, individual care plans); 3*. decision support*; evidence-based guidelines providing clinical standards for high-quality chronic care, and 4. development of *clinical information systems*; supplying care teams with feedback, reminding them to comply with practice guidelines and providing registries for planning individual and population-based care [7,15]. The two remaining components mainly refer to the context where chronic care is provided: 5. the *healthcare system *which encompasses the aforementioned elements, refers to the organizational context where chronic care is provided. A healthcare system seeking to improve chronic care must be motivated and prepared for change throughout the organization. Leadership must identify care improvement as important work, and translate it into clear improvement goals and policies that are addressed through application of effective improvement strategies and 6. links towards *community resources and policies*. The healthcare system is embedded in a community that includes organizations/programs that may support or expand a healthcare system's care for chronically ill patients (e.g. physical activity programs delivered by a local fitness centre). In our study, the term disease management was used for programs that included interventions that could be related to two or more components of the CCM.

### Data extraction

P4P schemes identified by the literature search were described on the basis of the nine dimensions of P4P schemes defined by Conrad and Perry [10] (Table 1). This classification was used to systematically disentangle the P4P schemes. We additionally reported the country where the scheme was introduced, the CCM elements to which the scheme could be related, and the goal of each scheme (Table 2). Of the papers reporting studies that evaluated the effects of P4P on quality of healthcare and healthcare costs we described the characteristics of the evaluation study (e.g. design, sample size, years of data collection), outcome measures, and study outcomes (Table 3).

**Table 1:** Features of P4P schemes and their dimensions

Feature	Dimensions
1. Type	• Reward: incentive implies increase in payments • Penalty: incentive implies decrease in payments

2. Nature incented entity	• Individual: incentive is granted to an individual (e.g. healthcare provider such as GP) • Group: incentive is granted to a group (e.g. clinical team, GP practice, hospital trust)

3. Focal quality behavior targeted by incentive	• Structure: incentives are based on resources assembled to deliver care (including personnel, facilities, IT, and materials) • Process: incentives are based on the completion of specific tasks or recommended treatments (e.g. performance measures, clinical quality) • Outcome: incentives are based on ultimate results of care (e.g. patient experience, health status, morbidity, and mortality)

4. Scope	• General: incentives target at general domain of quality (e.g. payment for each patient enrolled in disease management program). • Selective: incentives target a specific domain of quality (e.g. periodic blood pressure readings or cholesterol screening)

5. Motivation	• Intrinsic: incentive affects intrinsic motivation to deliver high quality care (e.g. patient benefit) • Extrinsic: incentive affects extrinsic motivation to deliver high quality care (e.g. economic interest)

6. Scale	• Relative: incentive is paid for achieving a given comparative ranking among providers (e.g. hospitals in top 2 performing quartiles are offered increases in tariff payments) • Absolute: incentives is paid for a continuous gradient of quality improvement (e.g. sickness funds receive higher payments for each patient enrolled in disease management program)

7. Size	• Amount of money provided or withdrawn

8. Certainty	• Certain: incented entity is certain about achievability of targets (e.g. targets seem easily achievable; guaranteed reward schedule) • Uncertain: incented entity is uncertain about achievability of targets (e.g. targets seem not easily achievable; competition for limited funds)

9. Frequency and duration	• Frequency: number of times a year an incentive is provided • Duration: number of years an incentive is provided

**Table 2:** General characteristics pay-for-performance schemes

Pay-for-performance scheme	Country	Elements chronic care model	Goal and patient population	Type	Incented entity	Focal quality behavior	Scope	Motivation	Scale	Size	Certainty	Frequency and duration
**Schemes in which financial incentives are granted to healthcare providers for delivering chronic care through disease management**												

Western New York Physician Incentive Program (WNY-PIP) [16]	USA	P4P simultaneously implemented with: 1. change in delivery system design (establishment of new routines in physician's office) 2. decision support (e.g. assessment tools, educating and training physician office staff, reflective information feedback ) 3. self-management support (e.g. providing patient education materials)	1. To improve chronic care treatment for diabetes patients 2. To explore effectiveness of financial incentives in improving care for this patient population 3. To promote new routines in physician's office Ultimate goal: improve patient health	Reward	Individual: Physician	Process: 6 clinical QI based on ADA clinical guideline Outcome: 3 patient outcome indicators	Selective: health plans pay financial incentives based on composite score on process and outcome indicators	N.A.	Absolute 1. scoring above predetermined target on composite score based on performance on process and outcome indicators 2. 50% improvement in composite score	Size of reward depends on weighted composite score. Actual payments varied from $3,000 till $12,000 (2003)	13 of 21 physician earned a financial reward.	Annually

Performance Based Incentive Program (PBIP) Highmark Blue Cross Blue Shield [12,17]	USA	P4P simultaneously implemented with: 1. decision support (e.g. up to date clinical physician guidelines, reflective information feedback, regular review by medical management consultant) 2. clinical information system (e.g. sharing practice specific data with other physicians for benchmarking)	Encourage healthcare providers to deliver best possible quality care and encourage coordinated care (patient population unknown)	Reward	Group: physician groups (not further specified)	Structure: electronic connectivity, Process: clinical quality Outcome: patient satisfaction	N.A.	N.A.	Relative: physician groups are rewarded if they exceed other physicians (in and out of the program) in performance on structure, process, and outcome indicators	N.A.	N.A.	N.A.

Partners Community Healthcare Inc./Brigham and Women's Physicians Organization pay-for-performance program (BWPO-P4P) [18,19]	USA	P4P simultaneously implemented with: 1. decision support (e.g. non-physician staff contact patients and physicians to improve compliance with practice guidelines) 2. change in delivery system design (e.g. central diabetes patient outreach coordinator tracking data and performance progress and checking compliance of patients; home visits) 3. clinical information system (adoption of electronic medical records and claims that track patient screening data (HEDIS) for e.g. benchmarking; central office sending reminder letters).	Improve quality and efficiency of care within the organization with regard to inpatient admissions, radiology, diabetes care, and asthma care. Only P4P scheme for diabetes and asthma care are relevant for our review.	Penalty: programs operate by withholding 10% of physician/hospital fees and returning those fees based on whether quality and efficiency targets are achieved	Group: network of primary care physicians, ophthalmologists, and staff	Process: clinical quality according to HEDIS measures Outcome: achieving target outcomes Shift from performance targets that focus on process indicators to targets that focus on outcome indicators	Selective: incentives based on performance on process and outcome indicators.	N.A.	Relative, withhold is returned if network: 1. scores above state or national 90^th ^percentile of HEDIS targets 2. improves baseline performance Some regional service organizations provide additional incentives directly to physicians whose patients meet HEDIS targets and many regional service organizations provide bonuses for non-clinical staff members who are critical to success of these programs	Portion of withholding that will be returned depends on performance on HEDIS measure (in 2006: moderate-volume primary care physician practices could earn additional $3000 to $5000 per physician if network met P4P HEDIS targets)	N.A.	Annually

Bridges to Excellence program (BTE) [12,20-23]	USA	P4P to stimulate implementation of: 1. self-management support (e.g. patient education, shared decision-making) 2. decision support (e.g. clinical standards set by NCQA/ADA) 3. clinical information system (e.g. adoption of electronic systems to maintain medical records documenting care delivery for reflective information feedback/benchmarking)	Create significant improvements in quality of asthma care, cardiac care, congestive heart failure care, coronary artery disease care, depression care, diabetes care, hypertension care, and spine care by recognizing and rewarding health care providers for implementing elements of CCM and delivering safe, timely, effective, efficient, equitable, and patient-centered care	Reward: higher revenue	Individual: physicians, nurse practitioners, and physician assistants certified through provider recognition program of NCQA	Structure: clinicians should comply with standards for clinical information systems Process: clinicians should comply with national standards for clinical care management, patient education and support	Selective: incentive based on whether healthcare providers meet a set of structure and process measures, which are scored to create overall program score where 60 is most often the passing grade.	N.A.	Absolute: incentive is provided when healthcare professionals meet certain performance measures. Each measure has an assigned maximum available point value. Clinicians achieve points for a measure based on the % of patient sample that meets or exceeds the set thresholds for that measure.	Depends on level of performance. Size of rewards changes over time and differs between health plans that participate in Bridges to Excellence.	N.A.	Annually

Integrated Healthcare Association Pay-for-performance Program (IHA-P4P) [24-29,31]	USA	P4P to stimulate implementation of: 1. change in delivery system design (e.g. redesigning processes and creating a systematic approach to diabetes care such as registries, actionable reports, and care management processes) 2. decision support (using evidence-based national measures) 3. clinical information system (e.g. adoption infrastructure for systematic processes of care; electronic medical records, reminder systems, reflective information feedback, benchmarking).	Stimulate provider organizations to consistently demonstrate high levels of quality performance with regard to preventive care, treatment of acute conditions, and treatment of chronic conditions (asthma, diabetes, and coronary heart disease) through public recognition and financial reward Only financial incentives for chronic conditions are relevant for our review.	Reward: provider groups earn financial rewards if they participate in the program and perform well on selected measures	Group: physician groups	Structure: adoption of IT enabled system to support patient care Process: 1. clinical quality: preventive screening, treatment of asthma, diabetes, and coronary artery disease; 2. coordinated diabetes care Outcome: patient experience Measure set is dynamic with new measures added each year.	Selective: health plans pay financial incentives based on composite score on established structure, process, and outcome measures. Composite score is calculated and then weighted: clinical quality 55%, patient experience 25%, coordinated diabetes registry 5%, IT enabled systemness 15%, resulting in overall performance score	N.A.	Absolute: Physician groups may receive incentive incremental financial payment for scoring in any of the category measures as long as the group scores in the appropriate percentile ranking as determined by the health plan.	Each health plan that participates in IHA-P4P scheme determines its own budget and methodology for calculating and distributing payments to physician groups. On average about 1% of base income of physician group (in 2009).	N.A.	Annually

Practice Incentive Program Diabetes Incentive (PIP-DI) [35-38]	Australia	P4P simultaneously implemented with: 1. self-management support (e.g. patient education in line with so-called diabetes and asthma cycles of care: minimum requirements to diabetes care based on national guidelines) 2. decision support (e.g. working in line with diabetes and asthma cycles of care), including support from regional primary care organization to encourage uptake 3. clinical information system (e.g. improvement of IT infrastructure)	To encourage GP's to effectively manage clinical diabetes and asthma care, mental health care and cervical screening. Only financial incentives for diabetes and asthma care are relevant for our review. Practices had to become accredited against standards of RACP.	Reward: incremental income	Group: GP practice	Structure: use of patient register and recall/reminder system Process: delivery of care according to national guidelines	Selective: incentives based on compliance with structure and process measures	N.A.	Absolute: incentive is provided when GP practices meet requirements	*Diabetes* $1 per standardized whole patient equivalent (SWPE) when using patient register and recall/reminder system (sign-on payment) $20 per patient to practices where at least 2% of patients are diagnosed with diabetes and GPs have completed a cycle of care for at least 20% of these patients (outcomes payment) $40 per patient per year for each completed cycle of care (service incentive payment) *Asthma *$0.25 per SWPE to practices that implement a cycle of care for patients with moderate to severe diabetes (sign-on payment) $100 per patient per year for each completed cycle of care for patients with moderate to severe asthma	N.A.	Quarterly Annually Quarterly Once Quarterly

Medicare Physician Group Practice Demonstration (MPGPD) [32,33]	USA	P4P simultaneously implemented with: 1. self-management support (e.g. patient education, active communication of patients with physicians and nurses, maintaining diet and exercise programs) 2. change in delivery system design (e.g. delegating tasks of physicians to non-physicians) 3. clinical information systems (e.g. electronic medical records, patient monitoring systems)	Quality improvement and cost efficiency of diabetes care, heart failure care, cardiac care, and preventive care at the level of the PGP	Reward	Group: PGP	Process: clinical quality according to HEDIS measures Outcome: clinical quality according to HEDIS measures 32 indicators are subset of CMS Quality Measurement and Health assessment Group for the Doctors Office Quality and comprise both process and outcome indicators.	Selective: Incentives based on performance on broad range of quality indicators which focused on diabetes mellitus, heart failure, coronary artery disease and hypertension, and preventive care.	N.A.	Absolute and relative Absolute: if cost saving ≥2% of target expenditures then 20% directly to Medicare and 80% to PGP. The portion provided to PGP is divided in cost performance payment (fixed payment) and quality performance payment. Quality performance payment is based on absolute and/or relative performance. To earn payment, PGP must achieve for each indicator at least 1 of 3 targets: 1. achieve ≥75% compliance or HEDIS mean for the measure (absolute); 2. achieve 70^th ^percentile Medicare level (relative); 3. demonstrate ≥10% improvement in compliance (absolute)	A shared savings provider payment model in which savings are shared between participating physician groups and the Medicare groups. A higher portion of the saving can be retained by PGP by good performance on indicators. Size depends on score on quality indicators. In total 2 PGPs received performance payments of in total $7.3 million as their share in the total saving of $9.5 million.	2 of 10 PGP earned a reward.	Annually

**Schemes in which financial incentives are granted to health insurers to enroll patients in disease management programs**												

Incentive to stimulate sickness funds to enroll patients in disease management program (DMP-P4P) [34]	Germany	P4P to stimulate implementation of certified DMPs. Information about when DMPs are classified as "certified" is limited. However, the following CCM elements are considered as important components of DMPs: 1. self-management support (e.g. patient education) 2. clinical information systems (e.g. quality management systems, feedback systems)	Stimulate sickness funds to enroll chronically ill patients (diabetes type 1 and 2, coronary heart disease, breast cancer, asthma, and COPD) in DMPs which are expected to improve quality and cost-effectiveness of healthcare for patients with chronic conditions	Reward: sickness funds that set op DMPs are rewarded with additional payments from risk adjustment scheme	Group: sickness funds	Structure: setting-up certified disease management program	General: if sickness funds set up certified DMPs and are able to enroll a high number of chronically ill patients for the relevant disease, they receive additional payments from risk adjustment scheme. If sickness funds do not set up DMPs or if they are able to do so but are not able to enroll a high number of chronically ill patients, they will receive fewer payments from the risk adjustment scheme	N.A.	Absolute: sickness funds receive higher payments for patients enrolled in certified DMP	Payments from risk adjustment system. Size unknown.	N.A.	N.A.

Notes: ADA = American Diabetes Association; CMS = Centers for Medicare and Medicaid Services; DMP = disease management program; HEDIS = Health Plan Employer Data and Information Set; N.A.= not available: information about these characteristics was not documented in the papers that we retrieved through our search process and/or could not be obtained from relevant websites; NCQA = National Committee for Quality Assurance; PGP = physician practice group; P4P = pay-for-performance; QI = quality indicators; RACP = Royal Australian College of General Practitioners.

**Table 3:** Effects pay-for-performance on healthcare quality

Incentive	Study design (N)	Year(s) data collection	Relevant outcome measures	Healthcare quality
Western New York Physician Incentive Program (WNY-P4P) [16]	Pre-post test Experimental group: patients (n = 624) whose physicians (n = 21) participated in P4P scheme Control group: sample of diabetic patients from health plan	2002-2003	• Quality of care based on a composite score which was based on process and outcome measures.	• Average of physician's composite scores increased 48% (baseline to end of project). • 13 out of 21 physicians improved their average composite score enough to earn some level of financial reward. • Of the 8 physicians not receiving reward, 6 improved their composite score.

Integrated Healthcare Association Pay-for-performance Program (IHA-P4P) [30]	Cross-sectional analysis of linked 2006 clinical performance scores from IHA-P4P and survey data from the 2^nd ^National Study of Physician Organizations among 108 California physician organizations.	2006	• Association between clinical performance and the use of chronic management processes • Association between clinical performance and electronic medical record capabilities • Association between clinical performance and participation in external quality improvement initiatives.	• Physician organizations investing more heavily in care management processes (e.g. patient registries, physician reminders and feedback, patient reminders and education) may achieve better performance scores. • Use of organized quality improvement efforts (e.g. participation in QI program) may be associated with increased delivery of recommended care processes, which in the context of the study translated into better performance on the clinical measures that were rewarded in the P4P scheme.

Practice Incentive Program Diabetes Incentive (PIP-DI) [38]	Retrospective study based on dataset from BEACH study (data from 100 consecutive encounters of 1000 GPs that are yearly randomly selected. Each encounter contains data on up to 4 problems treated, drugs prescribed, treatments conducted, referrals written and pathology). N = 12187: 1. Treatment group 1: accredited practices that use IT for internet, prescribing and medical records; 2. Treatment group 2: practices that are accredited and do not use IT for internet, prescribing and medical records; 3. Control group: practices that are not accredited and do not use IT, for internet, prescribing and medical records.	April 2002-March 2007 from	• Percentage of patients that received a glycosylated haemoglobin blood test during GP consult	• PIP-DI increased probability of a HbA1c test being ordered by 20 percentage points. • For patients from Aboriginal and Torres Straight Islander background the increase was more than 35 percentage points.

Practice Incentive Program Diabetes Incentive (PIP-DI) [37]	Descriptive study based on semi structured face-to-face interviews (22 GP practices)	2003	• Implementation of components of diabetes cycle of care	• Financial incentives may promote better clinical management. GPs claiming incentives were more likely to comply with all requirements than GPs that did not claim incentives.

External incentives (including financial incentives). [39]	Cross-sectional study: telephone survey among 1104 physician organizations (PO) with 20 or more physicians	2000-2001	• Extent of use of organized CMPs on the basis of summary measure: PO care management index, external incentives (bonus from health plans, public recognition, better contracts with health plans) quality reporting to outside organization (HEDIS data, clinical outcome data, results of quality improvement projects, patient satisfaction data), IT use	• External incentives and clinical IT were most strongly associated with CMP use. • Use of the most strongly associated incentives (public recognition and better contracts for healthcare quality) was associated with use of 1.3 and 0.7 additional CMPs (significant). • Receiving a bonus for scoring well on quality of care measures was not significantly associated with CMP use.

Notes: BEACH = Bettering the Evaluation and Care of Health; CMP = care management processes; FFS = fee for services; HEDIS = Health Plan Employer Data and Information Set; PO = physician organization; P4P = pay-for-performance; QI = quality improvement.

## Results

### Paper retrieval

**Figure 1:**
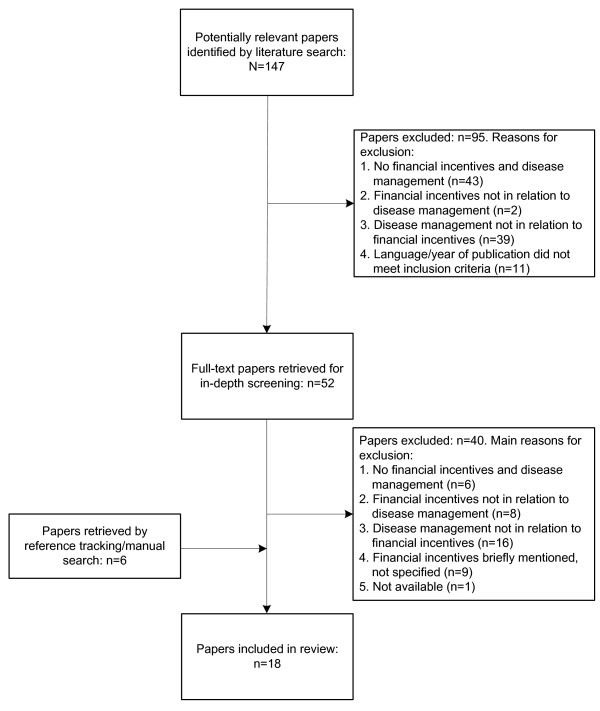
**Flow chart of literature screening process**.

Our literature search yielded 147 potentially relevant papers. On the basis of their title and abstract, 52 papers were selected to be retrieved full-text for in-depth screening. This screening process resulted into 12 papers eligible for our review. Reasons for exclusion are given in Figure 1. Additionally, six papers were identified through reference tracking and through a manual literature search on the internet. Hence, 18 papers were included in our review. The 18 papers described eight different P4P schemes that intend to improve chronic care delivery through disease management (Table 2). Some papers described the same P4P scheme which helped us to retrieve the fullest possible information about the different characteristics of the P4P schemes.

### Characteristics pay-for-performance schemes

#### General characteristics

Table 2 presents the characteristics of the eight included P4P schemes. Six P4P schemes were introduced in the USA: 1. the Western New York Physician Incentive Project (WNY-PIP) [16]; 2. Performance Based Incentive Program of Highmark Blue Cross Blue Shield (PBIP) [12,17]; 3. Partners Community Healthcare Inc./Brigham and Women's Hospital Physicians Organization pay-for-performance program (BWPO-P4P) [18,19]; 4. Bridges to Excellence Program (BTE) [12,20-23]; 5. Integrated Healthcare Association pay-for-performance program (IHA-P4P) [24-31]; and 6. the Medicare Physician Group Practice Demonstration (MPGPD) [32,33]. One P4P scheme was introduced in Germany: 7. Scheme to stimulate sickness funds to enroll patients in German disease management programs (further referred to as DMP-P4P) [34]. One P4P scheme was introduced in Australia: 8. Practice Incentive Program Diabetes Incentive (PIP-DI) [35-38].

We distinguished two categories of P4P schemes: 1. P4P schemes in which financial incentives are granted to healthcare providers for delivering chronic care through disease management. In these schemes, rewards are granted by health insurers, health plans or managed care organizations if healthcare providers deliver chronic care via disease management programs (WNY-P4P, PBIP, BWPO-P4P, BTE, IHA-P4P, PIP-DI, and MPGPD) and 2. P4P schemes in which financial incentives are granted to health insurers to motivate them to enroll patients in disease management programs (DMP-P4P). In this scheme sickness funds are rewarded by the German government for enrolling patients in disease management programs.

#### Elements of CCM

Five of the eight included P4P schemes were part of a larger scheme of interventions (i.e. quality improvement program) to improve quality of chronic care (WNY-PIP, PBIP, BWPO-P4P, PIP-DI, and MPGPD). In these schemes, P4P was simultaneously implemented with other interventions that can be related to elements of the CCM. Financial incentives were directed at healthcare providers to reward their participation in these schemes of quality improvement interventions. Three of the eight included P4P schemes were solely implemented (BTE, IHA-P4P, and DMP-P4P). In these P4P schemes, financial incentives are directed at healthcare providers or health insurers to stimulate them to implement interventions that can be related to elements of the CCM. In these schemes, financial incentives are granted after the implementation of interventions related to elements of the CCM.

Irrespective of the type of P4P scheme (i.e. P4P schemes part of a larger scheme of interventions vs. sole P4P schemes), we determined that the schemes stimulated delivery of chronic care through disease management by interventions that can be related to the same CCM elements. Interventions related to the CCM elements "decision support" (e.g. clinical guidelines, assessment tools) and "adoption of a clinical information system" (e.g. electronic medical records, reminder letters, sharing practice specific data for benchmarking and reflective information feedback) were observed in almost all P4P schemes. Self-management support interventions (e.g. patient education) were observed in WNY-P4P, PIP-DI, MPGPD, and DMP-P4P, whereas delivery system design interventions (e.g. home visits, patient outreach coordinator) were only observed in the WNY-P4P, MPGPD, and BWPO-P4P.

#### Goals

Generally, the goals of the P4P schemes were to stimulate delivery of high-quality and cost-effective chronic care, and ultimately to improve patient health. Most P4P schemes focused on delivery of integrated care for diabetes and/or asthma (WNY-P4P; BWPO-P4P; BTE; IHA; PIP-DI; MPGPD; DMP-P4P). Some P4P schemes additionally focused on care to other chronic patients including cardiac care (BTE; IHA-P4P; MPDGP), depression care (BTE), and COPD care (DMP-P4P), while of one P4P scheme the targeted patient population was unknown (PBIP).

#### Type

In seven of the eight P4P schemes, financial incentives were framed as rewards for delivering chronic care through disease management (WNY-P4P; PBIP; BTE; IHA-P4P; PIP-DI; MPGPD) or for enrolling patients in disease management programs (DMP-P4P). In one P4P scheme (BWPO-P4P), the financial incentive was framed as a penalty. Annual physician or hospital fees were withheld and were only returned if quality targets were achieved.

#### Incented entity

The incented entities differed over the eight included P4P schemes. In seven P4P schemes, financial incentives were granted to healthcare providers of which five were granted to groups of healthcare providers (e.g. physician groups, multidisciplinary groups of caregivers) (PBIP; BWPO-P4P; IHA-P4P; PIP-DI; MPGPD), whereas two were granted to individual healthcare providers (e.g. recognized physician, nurse practitioner, physician assistant) (WNY-P4P; BTE). In one P4P scheme, the financial incentive was granted to sickness funds (DMP-P4P).

#### Focal quality behavior

In most P4P schemes, financial incentives were granted on the basis of performance on a combination of structure (n = 6) and/or process indicators (n = 6). Performance on outcome indicators was less frequently used as a basis for granting financial incentives (n = 4). In one P4P scheme, the financial incentive was only based on performance on structure indicators (DMP-P4P). In two P4P schemes, financial incentives were based on performance on structure and process outcomes (BTE; PIP-DI). In three P4P schemes, financial incentives were based on performance on process and outcome indicators (WNY-PIP; BWPO-P4P; MPGPD) and in two P4P schemes, financial incentives were based on structure, process, and outcome indicators (PBIP; IHA-P4P).

Structure indicators were mostly based on the use of IT services (e.g. patient registries, recall/reminder system, electronic medical records) (e.g. BTE; PIP-DI) or having implemented a certified program (DMP-P4P). Process measures targeted at clinical quality (e.g. working according to national standards of clinical care management, patient education and support) and were often a subset of the Health Plan Employer Data and Information Set (HEDIS) (e.g. BWPO-P4P; BTE; IHA-P4P; MPGPD). Of the four financial incentives that were (partly) granted on the basis of performance on outcome measures, two P4P schemes targeted at patient experience/satisfaction (PBIP; IHA-P4P) whereas three financial incentives (WNY-P4P; BWPO-P4P, MPGPD) targeted at clinical outcome (i.e. % of patients with HbA1c value ≤ 7.5 or blood pressure ≤ 130/80).

#### Scope

Selective incentives were observed in six P4P schemes (WNY-P4P; BWPO-P4P; BTE; IHA-P4P; PIP-DI; MPGPD). These incentives targeted at specific domains of quality (e.g. periodic blood pressure readings, cholesterol screening, HbA1c screening) and were granted on the basis of scoring on established criteria of quality performance. A general incentive was observed in one P4P scheme (DMP-P4P). This incentive targeted at a general domain of quality, i.e. whether or not sickness funds set up disease management programs. The scope of one P4P scheme was unknown (PBIP).

#### Motivation

Information about whether the P4P-schemes affect intrinsic or extrinsic motivation to deliver high quality care was lacking in the papers that we included in our review. We therefore can not provide any information about this characteristic of the included P4P schemes.

#### Scale

Absolute performance incentives were observed in six P4P schemes (WNY-P4P; BTE; IHA; PIP-DI; DMP-P4P; MPGPD). Incentives were granted if healthcare providers or sickness funds meet established performance measures. Relative performance incentives were observed in three P4P schemes (PBIP; PIP-DI; MPGPD). Healthcare providers participating in these programs only received bonus payment if they exceeded performance of other healthcare providers.

#### Certainty

Five of the P4P schemes (WNY-PIP; BTE; IHA-P4P; PIP-DI; DMP-P4P) had guaranteed reward schedules, provided that established targets were achieved. Two studies illustrate by the number of physicians *not *receiving a reward that, despite the (partly) guaranteed reward schedules, targets established in the P4P schemes may not be easily achievable. A study evaluating the WNY-PIP showed that 13 out of 21 physicians earned a financial reward [16]. Also a study that evaluated the MPGPD, a scheme where rewards are granted on the basis of absolute and relative performance, showed that 2 out of 10 physician groups earned a financial reward [32].

In three P4P schemes (PBIP; BWPO-P4P; MPGPD), providing financial incentives was (partly) based on high relative performance. Rewarding only the top performers creates competition. This approach introduces uncertainty because a bonus depends not only on a healthcare providers' own performance but also on that of other healthcare providers in the network. Healthcare providers don't know in advance whether they will exceed competing healthcare providers.

#### Size, frequency, and duration

Of most P4P schemes, size, frequency, and duration of the financial incentives were not or only briefly described. Of six P4P schemes (WNY-PIP; PIP-DI; BWPO-P4P; BTE, IHA-P4P; MPGPD) some information was available about the incentive size. Of four of these incentives, the size depended on the composite performance score (WNY-P4P; BWPO-P4P; BTE; MPGPD). The precise size of rewards could change over time and could differ between the health plans that participated in the quality improvement programs. Health plans that participated in the IHA-P4P scheme determined their own budget and methodology for calculating and distributing payments to physician groups (IHA-P4P). Healthcare providers participating in PIP-DI received payment per standardized whole patient equivalent (IT structure) or per patient (that completed a cycle of care). No information was available about the size of the financial incentives granted via the PBIP and DMP-P4P.

### Effects pay-for-performance schemes

Of the 18 included papers, only five reported effects of P4P on healthcare quality [16,30,37-39], whereas no papers were found that reported the effects of P4P on healthcare costs. Of the five papers, four reported the effects of specified P4P schemes (WNY-PIP; PIP-DI; IHA-P4P) on healthcare performance, whereas one paper reported the effects of financial incentives in general versus the effects of other external incentives.

The evaluated aspects of healthcare delivery differed over the studies. The studies showed positive effects of P4P on the quality of care delivered. Scott et al. [38] revealed that PIP-DI increased the probability of a HbA1c test being ordered which implies a positive effect on quality of care in diabetes management. In the other study on PIP-DI, physicians were asked to what extent they implemented the nationally established minimum requirements to diabetes care. The study indicates that financial incentives promote better clinical management of diabetes patients: GPs claiming financial incentives were more likely to comply with all requirements than GPs that did not claim incentives [37]. Also the study that evaluated the effects of WNY-PIP on healthcare quality showed that the majority of the participating physicians improved their average score on process (i.e. screening of clinical parameters) and outcome (i.e. patient outcome) indicators [16]. The study evaluating the effects of IHA-P4P found that quality improvement efforts, such as P4P schemes, are positively related with improved delivery of clinical processes of care.

The study that evaluated the effects of external incentives, including financial incentives, did not find a significant relationship between receiving a financial reward for scoring well on quality of care measures between organized care management processes. However, significant relationships were found for other external incentives such as public recognition and better contracts for healthcare quality and organized care management processes [39].

## Discussion

This systematic literature review presents an overview of P4P schemes that are currently used to stimulate delivery of chronic care through disease management and provides insights into their effects. P4P schemes are increasingly implemented in the healthcare system to encourage improvements in healthcare quality. Well-known examples of such schemes are the Hospital Quality Incentive Demonstration Project introduced in the USA and the NHS Quality and Outcomes Framework and the Commissioning for Quality and Innovation Payment Framework introduced in the UK [12,21,40,41]. The increasing number of implemented P4P schemes has led to a mounting number of studies on the effects of such schemes. These studies have, however, not specified their results for P4P schemes that are used to stimulate delivery of chronic care through disease management [42-44]. The increasing number of disease management programs that have been implemented over the last years, has urged the need for insight into P4P schemes focusing on the implementation of disease management programs.

The included P4P schemes were systematically disentangled according to the dimensions of P4P schemes defined by Conrad and Perry [10]. This classification is in line with the characterization of P4P schemes of other authors who have recently published their work in this field [e.g. 12, 43, 45]. Systematically describing the P4P schemes facilitated comparison of their characteristics and effects. This process revealed that retrieving complete and up-to-date information about the characteristics of P4P schemes was difficult. In most papers schemes were not well-described and information given was often dated. Particularly, information about the certainty, motivation, size, frequency, and duration of the P4P schemes was difficult to obtain.

The included P4P schemes were rather similar in their goals (i.e. improving quality, continuity, and efficiency of care for the chronically ill) but differed in how to achieve these goals. Financial incentives targeting at healthcare providers strived for improvement of quality and continuity of care by stimulating healthcare providers to deliver chronic care through disease management, whereas incentives targeting at health insurers strived for improvement of quality and continuity of care by stimulating health insurers to enroll patients in disease management programs. We did not identify P4P schemes in combination with financial incentives for patients (e.g. rewarding patients for participating in disease management programs).

The included P4P schemes mostly stimulated delivery of chronic care through disease management by interventions that can be related to the CCM elements "decision support" and "clinical information system". This finding can possibly be explained by the fact that interventions related to these CCM elements are mostly implemented before interventions related to the other elements of the CCM [13,14].

Our literature review yielded only five papers that reported studies on the effects of P4P on healthcare quality [16,30,37-39]. No papers were found that reported studies evaluating the effects of P4P on healthcare costs. Most studies showed positive effects of P4P on the quality of care delivered. It should, however, be noted that the observed differences between schemes hinders comparability of their effects on healthcare quality and as a consequence drawing conclusions on the effectiveness of P4P to stimulate delivery of chronic care through disease management in general. It is therefore not possible to determine the characteristics of P4P schemes that may contribute to improved healthcare quality. This is in line with earlier studies that suggest that the effectiveness of P4P schemes is highly variable in terms of e.g. complications, ER waiting times, length of hospital stay, and screening rates and that the effectiveness depends on the design of the schemes and the characteristics of the context where they are introduced [11,43,44]. It should further be noted, that since our study mainly yielded studies showing positive effects of P4P schemes, publication bias should be taken into account. Since almost no studies were found showing no effects of P4P, it is difficult to determine the characteristics of P4P schemes that may not affect healthcare quality and healthcare costs.

Another issue which complicates drawing conclusions with regard to the effectiveness of P4P, which is also highlighted by other authors, is that P4P schemes are often not solely implemented [i.e. 19, 44]. Also the P4P schemes included in this study were mostly part of a larger scheme of interventions and were simultaneously implemented with other interventions than can be related to elements of the CCM such as patient registries, physician reminders and feedback, and the implementation of evidence-based guidelines and protocols. Such interventions also focus on changing healthcare providers' behavior and may interact with the potential effect of P4P. It is therefore difficult to determine the isolated effect of P4P on healthcare quality and related costs. Additionally, as also mentioned by Chaix-Couturier et al. [11] and Christianson et al. [44], contextual factors like the characteristics of the healthcare system where the P4P scheme is implemented (e.g. healthcare purchasing system, degree of regulation, financing mechanisms of the healthcare system), organizational aspects of the healthcare organization where the P4P scheme is implemented, and personal characteristics of the healthcare providers participating in the P4P scheme may also interact with the effect of the scheme and therefore further complicates comparability of the effects of P4P schemes. Hence, to interpret the results of the included studies it is also relevant to have information about the healthcare system where the P4P scheme is implemented and the simultaneously implemented interventions and their features.

Although the aforementioned issues complicate drawing conclusions with regard to which P4P schemes are most successful, there are some indications for the design characteristics of P4P schemes that may produce the largest effects. First, payment on the basis of scoring on process-based incentives may be more effective than indicators that are less directly related to a healthcare providers' performance [10,43]. Second, P4P schemes including a blend of individual- and group-level incentives may be more effective than P4P schemes only including individual or only including group-level incentives. Some performance issues can be improved most efficiently through group action, whereas others can be improved most efficiently through individual action [10,43,45]. Third, there are indications that P4P schemes rewarding absolute performance may be more effective than schemes rewarding relative performance. Rewarding relative performance may create uncertainty about the amount of additional revenue that can be obtained, because one's reward depends on the performance of other healthcare providers in the network. This, in turn, may result into unwillingness of providers to make investments in quality improvement. Moreover, only rewarding top performers may discourage healthcare providers with lower baseline quality to improve their services since for them it is difficult to outperform healthcare providers who already have high baseline quality [10,21,43,45]. It should, however, be noted that there are also indications that relative performance incentives are more effective in improving healthcare quality since they offer an ongoing incentive for initial high-quality providers to continue to perform well relative to their network [21].

On the basis of the current literature it is unclear whether these findings also apply to the design of P4P schemes to encourage delivery of care through disease management. Since evidence for the effectiveness of these P4P schemes is scarce, it is recommended to design methodologically sound studies to gain insight into the design characteristics that are most successful. Empirical evidence is necessary to carefully design P4P schemes and to ensure their effectiveness [10,42,43,45]. It is further recommended to determine the effect of P4P on equity in healthcare. A recent study of Doran et al. [46] suggests that the Quality and Outcomes Framework, implemented in the United Kingdom, has the potential to reduce inequalities in the delivery of primary clinical care. It is unknown if P4P schemes used to stimulate delivery of chronic care through disease management will produce similar effects. It is therefore recommended to include equity in healthcare as an outcome in future studies in this field.

When designing P4P schemes to stimulate disease management, it should further be taken into account that P4P schemes may also have negative consequences for the quality of care. A potential negative effect, also known as the "distortion effect", is the theoretical concern that stimulation of efforts on the measures of healthcare performance included in the P4P scheme may discourage efforts on aspects of healthcare performance that are not included and rewarded by the scheme. As a result, P4P may result into reduced healthcare quality, rather than into the intended improved healthcare quality [16,32]. Such potential side-effects of P4P should be included in future studies evaluating the effectiveness of P4P schemes.

## Conclusion

The number of P4P schemes intending to encourage delivery of chronic care through disease management is still limited. Hardly any information is available about the effects of such schemes on healthcare quality and healthcare costs.

## Competing interests

The authors declare that they have no competing interests.

## Authors' contributions

SdB and JS performed the literature search and selected relevant papers for the review. All authors analyzed the included papers. SdB drafted the manuscript and JS and CB revised the manuscript. All authors read and approved the final manuscript.

## Pre-publication history

The pre-publication history for this paper can be accessed here:

http://www.biomedcentral.com/1472-6963/11/272/prepub
